# Screening of Polyphenols and Antioxidative Activity in Industrial Beers

**DOI:** 10.3390/foods9020238

**Published:** 2020-02-23

**Authors:** Kristina Habschied, Ante Lončarić, Krešimir Mastanjević

**Affiliations:** Faculty of Food Technology Osijek, Josip Juraj Strossmayer University of Osijek, Osijek, F. Kuhača 20, 31000 Osijek, Croatia; ante.loncaric@ptfos.hr (A.L.); kmastanj@gmail.com (K.M.)

**Keywords:** polyphenols, antioxidative activity, HPLC, Folin-Ciocalteu, brown glass bottle

## Abstract

Antioxidative molecules, such as polyphenols can preserve and prolong the freshness of packaged beers. The aim of this work was to assess the content of polyphenolic compounds (by Folin-Ciocalteu and standard European Brewery Convention method) in different types of industrially produced beers (lager, pilsner, black and dark), packaged in brown glass bottles. The results of this research indicate that there are significant changes in polyphenol concentrations in correlation with beer type. Polyphenolic content was highest in dark and black beers. Antioxidative activity was also more pronounced in dark and black beers. Most prominent phenolic acid in all samples was gallic acid. Two samples of dark and three samples of black beers had >10 mg/100 mL of this polyphenol, with maximal value of 14.22 mg/100 mL in sample CK (black beer). This would indicate that black beers are richer in polyphenolic content than the light (lager and pilsner) beers and the moderate consumption of such beer could contribute to the health of consumers.

## 1. Introduction

Polyphenol content in beer differs in regards to the type of malt and hops used in production. About 80% of polyphenols in beer originate from malt or other cereals added to mash, malted or unmalted, and 20% originates from hops (*Humulus lupulus*). The ratio is dependent on beer type [1,2]. Phenolic compounds often appear in the form of esters, glycosides, and may be bound to complex compounds, such as polysaccharides [3]. 

Beer is rich in different groups of polyphenolic compounds from which tannins, phenolic acids, flavones, and flavonols are the most prominent. Xanthohumol and related prenylflavonoids (originated from hop) are also important for beer flavor and aromas [4]. This is especially pronounced in dark beers [3,5]. All these compounds have an important role in beer taste and aroma formation. Beer polyphenols contribute to other important characteristics of beer: astringency, body and fullness [5,6]. Flavonoids in beer, responsible for astringency (cathechin and epicatechin) are desirable in concentrations 1–20 mg/L [7]. Beers with pH 4–4.2 have a more pronounced astringency. Flavonoids contribute to bitterness of beer but the basic bitterness comes from hops α-acids [3]. 

Phenolic components of beer represent great interest for brewers since they directly affect the quality of beer. Besides their positive effect on preventing oxidation, they can negatively influence the colloidal and foam stability and, thus shorten the shelf life of beer [8,9]. Some reports, however, consider polyphenolic compounds as one of the quality indicators for beer processing and marketing [10].

Recent studies correlate the consumption of polyphenols-rich foods with the prevention of many modern diseases associated with oxidative stress [11,12,13,14]. Even though ethanol has been regarded as carcinogenic to humans (WHO IARC group 1) [15] a mild or moderate ethanol consumption in the form of beer or wine can contribute to the overall positive status of human health [16,17,18,19,20].

The antioxidative activity also depends on the constituents of beer. For that reason, the aim of this research was to assess the physical-chemical properties of different styles of beer packaged in brown glass bottles and to report the differences between polyphenolic content and antioxidative activity determined in them. The intention of this research was also to give an overview of polyphenolic compounds in beers which can be found on the Croatian market. Since no similar research has been conducted in Croatia, this survey gives an important insight in different types and beer styles found in supermarkets. 

## 2. Materials and Methods

### 2.1. Materials

Caffeic acid (C0625, 99%), p-coumaric acid (C9008, ≥98%), gallic acid (27645, ≥99%), epicatechin (E1753, ≥90%), 2,2-diphenyl-1-picryl-hydrazyl radical (DPPH, D9132), Carrez Reagent I (MAK191A), Carrez Reagent II (MAK191B) were purchased from Sigma-Aldrich (St. Louis, MO, USA). Orto-phosphoric acid 85%, HPLC grade, methanol HPLC grade, hydrochloric acid 36.2% were purchased from Fluka (Buchs, Switzerland) and sodium carbonate and Folin-Ciocalteu reagent from Kemika (Zagreb, Croatia).

### 2.2. Beer Samples

Samples of twelve commercially available beer brands, from producers in Croatia, Germany, and Czech Republic were bought at the supermarket. Every sample was bought in triplicate, with special care of buying beer from the same batch and with the identical shelf life. Four beer styles were chosen to be analyzed: lager, pilsner, dark and black beer. The samples were stored in a dark and cool place at 4 °C until further analysis. The list of the conducted analysis is shown in Table 1. All analyses were done in triplicate, according to the standard MEBAK^®^ (Mitteleuropeaschie Brautechniche Analysenkommission) and European Brewing Convention (Analytica-EBC^®^) methods [21,22]. 

### 2.3. Determination of Total Polyphenolic Index

The total polyphenolic index (TPI) was determined by employing Folin-Ciocalteu reagent (FC) according to a procedure described by Singleton and Rossi [23]. The changes in the color of radical from light blue to dark blue were measured after 30 min at 760 nm using a UV-ViS spectrophotometer (Shimadzu UVmini-1240, Kyoto, Japan). The TP was quantified from gallic acid calibration curve (3–20 mg/L, *R*^2^ = 0.9961). The TPI was calculated and expressed as mg gallic acid equivalent (GAE) per L of beer.

Total polyphenols were also determined and quantified using standard spectrophotometric Analytica EBC® Method 9.11 [21].

### 2.4. Determination of Antioxidant Activity

The antioxidant activity was measured using a DPPH radical according to modified method described by Brand-Williams et al. [24]. The reaction mixture consisted of 0.2 mL of beer sample and 3 mL of DPPH radical solution 0.1 mM in methanol. The changes in the color of radical from deep violet to light violet were measured after 30 min at 515 nm using a UV-ViS spectrophotometer (Shimadzu UVmini-1240, Kyoto, Japan). The antiradical activity (AA) was determined using the following equation (y = 0.9548x + 0.0294; *R*^2^ = 0.9914) obtained from linear regression after plotting the A515 nm of known solutions of trolox against concentration (0.1–0.9 mM). The results were expressed as milliomoles of Trolox® equivalents (TE) per one liter of beer (mmol TE/L).

### 2.5. Polyphenols Analysis via High-Performance Lliquid Chromatography with Ddiode-Array Detector (HPLC-PDA)

Polyphenols were identified and quantified by Varian LC system (Agilent, Palo Alto, CA, USA) equipped with a ProStar 230 solvent delivery module, mand ProStar 330 PDA Detector. Polyphenols separation was done in an OmniSpher C18 column (250 × 4.6 mm inner diameter, 5 µm, Varian, Atlanta, GA, USA) protected with guard column (ChromSep 1 cm × 3 mm, Varian, Atlanta, GA, USA) using 0.5% water solution of phosphoric acid as a solvent A and 100% HPLC grade methanol as a solvent B (elution conditions: 0–38 min from 3 to 65% B; 38–45 min, 65% B; flow rate 1 mL/min, injection volumes were 20 mL). For each sample, three replicated HPLC analyses were performed, and the results were given as mg per L of beer (mg/L). UVeVis spectra were recorded in wavelength range from 190 to 600 nm. Caffeic acid and *p*-coumaric were detected at 320 nm while gallic acid and epicatechin were detected at 280 nm. Quantification was performed by external standard calibration. Standards (gallic acid, *p*-coumaric acid, epicatechin) were purchased from Sigma-Aldrich (Munich, Germany). Samples were pre-treated by degassing and mixing. Removing of matrix interfering compounds (such as proteins, redox compounds, etc.) was carried out using Carrez’s solutions. Two mL of sample and 200 μL of each Carrez’s solution were added in a 5 mL volumetric flask and the volume was completed with water. The mixture was homogenized, allowed to stand for 15 min before filteration through filter paper (Whatman No. 1). Prior to HPLC injection, samples were filtered through a cellulose ester membrane (0.22 μm). 

### 2.6. Statistical Analysis

Statistical analysis was carried out using Statistica Ver. 8.0 StatSoft Inc. Tulsa, OK, USA. Data analysis: differences between the average values of polyphenolic content and antioxidative activity analyzed using the analysis of variance (ANOVA) and the Fisher’s least significant difference test (LSD), with statistical significance being set at *p* < 0.05. The results for polyphenolic content determined by both methods, Folin-Ciocalteu (mg/L) and EBC (mg/L), and antioxidatve activity (mmol Trolox/100 mL) were subjected to correlation analysis (Pearson’s correlation test) in order to determine their possible statistically meaningful relationships.

## 3. Results and Discussion

The results of conducted analysis are presented in Table 2 and Table 3. The obtained results presented in Table 2 show the values of different basic quality indicators of beer. 

Quality indicators such as bitterness, color and foam stability are important for consumers and have to remain constant quality in every batch. Quality indicators also reveal much on the beer style, especially color. Table 2 shows significant increase of color for dark and black styles of beer, in comparison to pilsners and lagers. The usage of colored malt, which is a result of higher temperatures during kilning, significantly influences beer color. Color influences beer acceptance among consumers since darker beers usually indicate heavier and fuller beers, which are customary in countries with colder climate. Bitterness is one of the most important qualities of beer, and is very important for the consumer. Da Costa Jardim et al. [25] conducted a research on the relationship between polyphenols, bitterness and composition of main craft beer styles and consumer preference for them. The results they presented indicate that the preferred beer style was the one that is less bitter. A less bitter beer usually involves lower polyphenolic content [25]. However, this was not the case in our research since some of the samples exhibited higher bitterness (3SB and 5SZ, Table 2) but phenolic content was lower than in black or dark beers (9TB and 11CCK) with higher phenolic content (Table 3). This could be because of the caramel taste in black and dark malts masks the hoppy bitterness. 

Original and apparent extract are indicators of fermentation course. Original extract is the percent of solids (*w*/*w*) that can be found in unfermented wort, expressed as % sucrose. Real extract describes the amount of solids in final beer. They are important for brewers because they help monitor the produced alcohol in order to respect the government regulations regarding the taxes for produced alcohol, as the alcohol content is closely related to the real extract and specific gravity. Even though majority of beers had similar original extract content, around 11 °Plato (except 8TK who had <10 °Plato and 11CCK and 12CK who had >12 °Plato), bitterness, narrowly related with the polyphenolic content in beer, showed variations between styles. Socha et al. [3] tried to correlate extract content with the antioxidative activity of dark beers, but the investigation showed that original extract is not a reliable source of information regarding antioxidant content and activity of dark beers. This appeared to be the case in our research too. 

Alcohol content was determined in all beer samples and was in accordance with the beer style preferences. Pilsners, lagers, darks and black beers were mostly below 5 *v*/*v* with the exception of samples 5SZ (5.14 *v*/*v*), 7TP (5.30 *v*/*v*) and 12CK (6.04 *v*/*v*). Sample 8TK, belonging to dark beers, had exceptionally low alcohol content, 3.67 *v*/*v* which is in accordance with the lower original extract value, 9.75 °P. 

Polyphenols are reported as important factors in foam (in) stability. Namely, some authors report a positive correlation between foam stability (head retention) and polyphenols in beer [26], while others reported a negative correlation [27]. It is hard to pinpoint the actual effect of polyphenols on beer foam since in the industrial production of beer foam promoting additives (i.e., propylene glycol alginate esters) can be added in order to retain the head for a longer time [28]. Also, the high foam stability was detected in pilsner beers, ranging from 8 to 10 min. Lager heads were also stable, with minimal value of 5 min and maximal of 8 min. Dark beers showed similar trend, but in sample 8TK no foam was detected. Black beers also showed high foam stability with the exception of sample 11CCK in which no foam was noted upon pouring it down the glass. However, no clear correlation could be determined between the foam stability and polyphenol content in this research.

Polyphenolic content was measured using standard EBC method and Folin-Ciocalteu method and the results are shown in Table 3. According to the EBC method, only two samples, one dark (9TB) and one black (11CCK) showed polyphenolic content higher than 200 mg/L. Pilsner and lager styles showed polyphenolic values 98–134 mg/L, where pilsners had generally lower values of polyphenols.

Folin-Ciocalteu method quantified 3–5 times higher values of polyphenols than the EBC method. This is in accordance with a research conducted by Kalušević et al. [29]. Bendelov [30] compared the EBC method and an automated method using gallic acid. He established a correlation between the two methods, 0.88 for lighter beers and 0.92 between the values for darker beers. Pearson’s correlation test applied to the samples in this study (Table 4) established a somewhat lower correlation factor between the two methods, *r* = 0.77. However, the correlation between both methods (Folin-Cocalteu and EBC) and antioxidative activity showed higher (*r* = 0.92) correlation between antioxidative activity and EBC method thus making it more suitable and reliable for polyphenolic content determination.

In our research, a significant difference was noted between values obtained with different methods for the same samples. According to general observation, light beers had lower polyphenols concentration than dark and black beers. Highest concentration of polyphenols in light beers was detected in pilsner type of beer, sample 2SS with 579 mg/L. The results measured using EBC method have showed highest levels of polyphenols for the same sample, 134 mg/L. However, other results did not show similar correspondence between methods. The highest concentration of polyphenols in dark and black beers, determined using Folin-Ciocalteu method, appeared to be found in the sample, 11CCK (855 mg/L), followed by sample 12CK with 850 mg/L. Folin-Ciocalteu method was assessed and described in many papers regarding beer polyphenols [31,32,33]. Even though the Folin-Ciocalteu method is used for beverages or plant extracts analysis, it does not respond only for phenolic compounds and the interference with other compounds may cause overestimation of results [34]. The results always show higher concentrations, in comparison to the EBC method. The phenolic concentrations show an increase for concentrations of different compounds with reducing activity, such as Maillard reaction products, sulfites or other similar substance. This is why the identification and quantification of individual phenolic compounds (Table 3) is important for true phenolic profile of beer [32]. 

Gallic acid belongs to hydroxybenzoic (phenolic) acids. According to Socha et al. [3] gallic acid can be a good indicator of the oxidation processes during beer production because of its high susceptibility to oxidation and degradation. This group also involves *p*-coumaric and caffeic acids, characterized as hydroxycinnamic acids. Phenolic acids do not affect the flavor and odor, but they act as precursors for volatile phenolic compounds. Phenolic compounds oxidize very easily when they come in touch with oxygen and in oxidized state they may interfere the sensory properties such as flavor, aroma and color [35]. 

Gallic, caffeic and *p*-coumaric acids were quantified because they exhibited significant concentrations in previous research on polyphenolic constituents in beer [10,32,36,37,38]. From the results in Table 3 it can be noted that caffeic acid is found in tested beers in lowest concentration when compared to other phenolic compounds. *P*-coumaric acid and epicatechin follow while gallic acid was quantified as the most abundant. Highest levels of gallic acid were detected in a sample of black beer 12CK (14.22 mg/100 mL), while the lowest concentration was found in sample 6SK (4.12 mg/100 mL), a light lager style beer. Similar values were reported by Zhao et al. [32]. Light and dark beers (samples 1–9) had the lowest share of *p*-coumaric acid and higher epicatechin share. Black beers (samples 10–12) had higher concentrations of *p*-coumaric acid and lower concentration of epicatechin. Mitić et al. [10] reported somewhat lower levels for gallic acid in dark beers, but it was still the most abundant polyphenol in all samples. 

Antioxidative activity (AA) was detected in all beers, but it was more pronounced in dark and black beers. For pilsners and lagers it ranged from 0.40 (mmol trolox/100 mL) for sample 1SW to 0.5 (mmol trolox/100 mL) for sample 5SZ. Dark and black beers showed higher values for antioxidative activity with the lowest value of 0.54 (mmol trolox/100 mL) for dark sample 7TP and 0.61 (mmol trolox/100 mL) for black beer sample 12CK. These results correlate well with the polyphenolic content for both methods. The results for antioxidative activity obtained in our research can be compared with the result published by Socha et al. [3]. According to Marova et al. [4], beer consist of two major groups responsible for antioxidative activity: hop flavonoids (e.g., xanthohumol and related prenylflavonoids); and malt phenolics (e.g., phenolic acids and flavonoids). Socha et al. [3] reported that the observed variability of AA for different dark beers must be related to the differences in raw materials (i.e., barley and hop) and malting (higher temperatures during kilning) and brewing (contact with O_2_, boiling time and temperature, maturing time and equipment) processes. The addition of citric acid (or other compounds exhibiting reducing activity) as a preservative in some beers, can also affect the AA values of this research. 

## 4. Conclusions

In conclusion, polyphenols are very stable molecules and can be found in various concentrations in different beer styles. According to our research the most popular beer styles in our region, lager and pilsner showed lower polyphenolic content and lower antioxidative activity than dark and black beers. Identification and quantification of individual phenolic compounds showed that phenolic compounds can be found in dark and black beers in higher concentrations. Health-wise, this would indicate that dark beers are more beneficial for overall human health, but only in moderate volumes (cca. 0.5 L per day). Two different methods for polyphenolic content analysis, Folin-Ciocalteu and EBC, correlated well with antioxidative activity but EBC showed higher correlation factor, which makes it more suitable for polyphenolic content determination in matrix such as beer.

## Figures and Tables

**Table 1:** Methods and the abbreviations used for the analysis of physical-chemical quality indicators in chosen samples.

Indicator	Unit	Method	
		MEBAK^®^	(Analytica-EBC^®^)
Original Extract	°Plato	2.13.16.1	
Apparent Extract	°Plato	2.13.3	
Specific Gravity	g/mL	2.13.16.1	
Alcohol by volume	mL/100 mL	2.13.16.1	
pH value		2.17.	
Color	EBC	2.16.2	
Bitterness	BU	2.22.1	
Polyphenols	mg/L		9.11.
Foam stability	min	2.23.1	

**Table 2:** Different beer style characteristics from different Country of origin.

Sample	Country	Beer Style	Original Extract (°Plato)	Apparent Extract (°Plato)	Specific Gravity (g/mL)	Alcohol Content (*v*/*v*)	pH	Color (EBC)	Bitterness (EBC)	Foam Stability (min)
1SW	Germany	Pilsner	11.05	3.79	1.0082	4.75	4.3	8	24	10
2SS	Croatia	Pilsner	11.63	4.41	1.0106	4.76	4.6	7	19	8
3SB	Germany	Pilsner	11.11	3.90	1.0086	4.72	4.3	7	28	10
4SO	Croatia	Lager	11.51	4.40	1.0104	4.67	4.3	6	17	8
5SZ	Croatia	Lager	11.90	4.09	1.0088	5.14	4.3	12	30	7
6SK	Czech	Lager	11.07	3.95	1.0089	4.66	4.6	12	24	5
7TP	Croatia	Dark	11.86	3.79	1.0074	5.30	4.2	44	14	7
8TK	Czech	Dark	9.75	4.12	1.0109	3.67	4.5	76	13	- *
9TB	Germany	Dark	11.75	4.59	1.0114	4.72	4.5	82	21	8
10CO	Croatia	Black	11.90	4.66	1.0116	4.77	4.4	81	23	10
11CCK	Croatia	Black	12.97	5.46	1.0144	4.80	4.4	88	19	- *
12CK	Croatia	Black	14.36	5.35	1.0126	6.03	4.3	70	22	11

* no foam detected; values are means of three measurements ± SD.

**Table 3:** Content of polyphenols and antioxidative activity of analyzed samples.

Sample	Gallic Acid (mg/100 mL)	Caffeic Acid (mg/100 mL)	*p*-Coumaric Acid (mg/100 mL)	Epicatechin (mg/100 mL)	Polyphenols Folin—Ciocalteu (mg/L)	Polyphenols EBC Method (mg/L)	Antioxidatve Activity (mmol Trolox/100 mL)
1SW	4.17 ± 0.01 ^j^	0.55 ± 0.02 ^f^	0.36 ± 0.03 ^i^	1.45 ± 0.01 ^gh^	480.30 ± 0.29 ^j^	98.02 ± 0.31 ^j^	0.40 ± 0.01 ^e^
2SS	8.66 ± 0.03 ^f^	1.33 ± 0.02 ^d^	1.07 ± 0.18 ^g^	2.62 ± 0.06 ^e^	579.29 ± 0.34 ^e^	134.48 ± 0.71 ^e^	0.48 ± 0.06 ^cd^
3SB	5.12 ± 0.01 ^h^	0.93 ± 0.02 ^e^	0.37 ± 0.01 ^i^	1.64 ± 0.02 ^g^	498.89 ± 0.27 ^h^	100.36 ± 0.62 ^i^	0.47 ± 0.03 ^cd^
4SO	5.04 ± 0.02 ^i^	0.76 ± 0.04 ^ef^	0.36 ± 0.04 ^i^	1.42 ± 0.03 ^gh^	488.38 ± 0.16 ^hi^	112.67 ± 0.33 ^g^	0.46 ± 0.01 ^d^
5SZ	7.65 ± 0.01 ^g^	1.17 ± 0.08 ^d^	1.53 ± 0.03 ^f^	2.34 ± 0.09 ^f^	539.49 ± 0.13 ^g^	102.48 ± 0.47 ^h^	0.50 ± 0.01 ^c^
6SK	4.12 ± 0.01 ^k^	0.87 ± 0.04 ^e^	0.71 ± 0.02 ^h^	1.26 ± 0.03 ^h^	464.34 ± 0.06 ^j^	114.78 ± 0.83 ^f^	0.40 ± 0.01 ^e^
7TP	8.97 ± 0.02 ^e^	1.56 ± 0.33 ^c^	1.04 ± 0.04 ^g^	2.67 ± 0.22 ^e^	563.54 ± 0.18 ^f^	115.27 ± 0.70 ^f^	0.54 ± 0.01 ^b^
8TK	11.25 ± 0.03 ^d^	2.07 ± 0.02 ^b^	1.87 ± 0.02 ^e^	3.25 ± 0.09 ^d^	708.38 ± 0.07 ^d^	160.12 ± 0.52 ^c^	0.59 ± 0.00 ^a^
9TB	13.46 ± 0.02 ^c^	2.10 ± 0.00 ^b^	2.23 ± 0.11 ^d^	3.44 ± 0.20 ^cd^	776.46 ± 0.21 ^b^	259.90 ± 0.23 ^a^	0.60 ± 0.01 ^a^
10CO	11.22 ± 0.02 ^d^	2.35 ± 0.28 ^a^	3.45 ± 0.04 ^c^	3.67 ± 0.37 ^c^	723.33 ± 0.09 ^c^	141.89 ± 0.76 ^d^	0.58 ± 0.00 ^a^
11CCK	14.05 ± 0.03 ^b^	2.29 ± 0.09 ^ab^	5.26 ± 0.05 ^b^	4.55 ± 0.04 ^a^	855.45 ± 0.06 ^a^	235.23 ± 0.98 ^b^	0.60 ± 0.00 ^a^
12CK	14.22 ± 0.04 ^a^	2.25 ± 0.01 ^ab^	5.58 ± 0.18 ^a^	4.30 ± 0.07 ^b^	849.50 ± 0.13 ^a^	140.17 ± 0.28 ^d^	0.61 ± 0.00 ^a^

Values are means of three measurements ± SD. Values are means of three measurements. Values in the same column with different letters a–d or in same row (a–h) are significantly different (*p* < 0.05).

**Table 4:** Pearson’s correlation coefficients.

	Polyphenols Folin—Ciocalteu (mg/L)	Polyphenols EBC (mg/L)	Antioxidatve Activity (mmol Trolox/100 mL)
Polyphenols Folin—Ciocalteu (pF-C) (mg/L)	1	0.77 **	0.69 **
Polyphenols (EBC) (mg/L)	0.77 **	1	0.92 **
Antioxidatve activity (AA) (mmol Trolox/100 mL)	0.69 **	0.92 **	1

Values marked with ** are statistically significant (*p* < 0.05).
